# Acute care nurses’ perceptions of leadership, teamwork, turnover intention and patient safety – a mixed methods study

**DOI:** 10.1186/s12912-021-00652-w

**Published:** 2021-07-30

**Authors:** Shahram Zaheer, Liane Ginsburg, Hannah J. Wong, Kelly Thomson, Lorna Bain, Zaev Wulffhart

**Affiliations:** 1 School of Health Policy and Management, York University, Toronto, Canada; 2 Daphne Cockwell School of Nursing, Ryerson University, Toronto, Canada; 3 Lawrence S. Bloomberg Faculty of Nursing, University of Toronto, Toronto, Canada; 4 School of Administrative Studies, York University, Toronto, Canada; 5 Interprofessional Collaboration and Education, Southlake Regional Health Centre, Newmarket, Canada; 6 University of Toronto, Toronto, Canada; 7 Regional Cardiac Care Program, Southlake Regional Health Centre, Newmarket, Canada

**Keywords:** Patient safety, Senior leadership, Supervisory leadership, Teamwork, Turnover intention

## Abstract

**Background:**

This study contributes to a small but growing body of literature on how context influences perceptions of patient safety in healthcare settings. We examine the impact of senior leadership support for safety, supervisory leadership support for safety, teamwork, and turnover intention on overall patient safety grade. Interaction effects of predictors on perceptions of patient safety are also examined.

**Methods:**

In this mixed methods study, cross-sectional survey data (*N* = 185) were collected from nurses and non-physician healthcare professionals. Semi-structured interview data (*N* = 15) were collected from nurses. The study participants worked in intensive care, general medicine, mental health, or the emergency department of a large community hospital in Southern Ontario.

**Results:**

Hierarchical regression analyses showed that staff perceptions of senior leadership (*p* < 0.001), teamwork (*p* < 0.01), and turnover intention (*p* < 0.01) were significantly associated with overall patient safety grade. The interactive effect of teamwork and turnover intention on overall patient safety grade was also found to be significant (*p* < 0.05). The qualitative findings corroborated the survey results but also helped expand the characteristics of the study’s key concepts (e.g., teamwork within and across professional boundaries) and why certain statistical relationships were found to be non-significant (e.g., nurse interviewees perceived the safety specific responsibilities of frontline supervisors much more broadly compared to the narrower conceptualization of the construct in the survey).

**Conclusions:**

The results of the current study suggest that senior leadership, teamwork, and turnover intention significantly impact nursing staff perceptions of patient safety. Leadership is a modifiable contextual factor and resources should be dedicated to strengthen relational competencies of healthcare leaders. Healthcare organizations must also proactively foster inter and intra-professional collaboration by providing teamwork educational workshops or other on-site learning opportunities (e.g., simulation training). Healthcare organizations would benefit by considering the interactive effect of contextual factors as another lever for patient safety improvement, e.g., lowering staff turnover intentions would maximize the positive impact of teamwork improvement initiatives on patient safety.

**Supplementary Information:**

The online version contains supplementary material available at 10.1186/s12912-021-00652-w.

## Background

Health care delivery systems suffer from a variety of quality problems such as underuse, overuse, and misuse of health services. There are a variety of causes of these quality problems including rapid advancements in medical technologies, aging populations with comorbid chronic illnesses, complex care processes etc. These interrelated factors also contribute to complexity and tight coupling among system components that can eventually lead to medical errors. Empirical evidence from a number of international studies suggests that an adverse event occurs in up to 10% of hospitalizations and that half of these events are preventable [1–3]. Moreover, medical errors are costly; for example, preventable adverse events in Canadian acute care systems result in $397 million in extra health care costs annually [4]. Over the last decade, implementation of standardized clinical interventions such as hand hygiene guidelines and surgical checklists have reduced preventable medication, diagnostic and surgical errors [5]. However, a growing body of evidence suggests that contextual factors (e.g., teamwork and culture) positively influences perceptions of patient safety [6] and can increase the likelihood of successfully implementing these safety improvement interventions [7, 8].

The objectives of this mixed-methods study are to examine the relationships (i.e., direct and moderated) between nurses’ perceptions of senior leadership, supervisory leadership, teamwork, turnover intention and a self-reported patient safety measure. Further evidence of the relationship between contextual factors, such as leadership support for safety and teamwork, and outcomes such as patient safety can contribute to a growing body of empirical work on the role of context in improving quality and safety practices.

### Relational factors affecting perceptions of patient safety

Empirical evidence in healthcare settings suggest that safety climate perceptions of employees can be significantly improved by leaders’ safety related behaviours such as frontline safety forums [9], senior leadership walkrounds [10], adopt-a-work unit [11] establishing unit norms of openness [12], and adopting situation specific leadership style [13]. The positive impact of leadership support for safety on patient outcomes (e.g., decreased falls, lower rates of medication errors, and less likelihood of hospital-acquired infections) is also starting to emerge in healthcare research [14–16]. However, only a handful of empirical studies have examined the interactive effect of senior and supervisory leadership on safety outcomes [6, 17]. There is a need for further empirical research to better understand the impact of different levels of leadership on patient safety.

In the past, highly specialized professionals operating in silos were often sufficient to provide appropriate treatment to patients. However, changing disease patterns and growing complexity of care delivery now require healthcare teams to engage in teamwork behaviours (e.g., communication, coordination, cooperation) to reduce preventable errors and improve safety outcomes [18]. Indeed, emerging empirical evidence suggests that positive staff perceptions of teamwork are associated with better patient safety outcomes – e.g., reduced odds of poor surgical outcomes [19], reduced incidence of in-hospital adverse events [14] and reduced hospital readmission rates [20].

Some employee turnover is to be expected, however, safe functioning of healthcare organizations is threatened when workforce turnover is high [21]. There are direct (e.g., hiring, advertisement, and recruitment costs) and indirect (e.g., lower morale, reduced productivity, increased workload) negative consequences of high employee turnover rate on organizational functioning and performance [22]. High turnover can trap an organization in a vicious cycle where remaining employees are more likely to leave due to increased workload and low morale [21, 23]. In healthcare organizations, the well-being of patients is at risk when employee turnover is high. For example, healthcare-acquired infections, hospitalizations, and medical errors are more likely in the presence of high nursing turnover [24, 25]. On the other hand, occurrence of medication errors, patient falls, and adverse events are less likely when nursing turnover is low [26]. Turnover intention is the strongest most immediate predictor of turnover and hence a valid proxy for employee leaving behaviours [22, 27]. Turnover intention is also likely to be associated with many of the same indirect costs as turnover (e.g. low morale, reduced workforce productivity). However, to our knowledge, none of the previous empirical studies have examined the relationship between healthcare staff turnover intention and their perceptions of patient safety outcomes. There is a need to empirically examine this pertinent relationship due to conceptual (e.g., supporting turnover intention as a valid proxy of turnover) and practical (e.g., encouraging healthcare organizations to proactively implement staff retention strategies) implications.

The research community has made important inroads in understanding the impact of context-related predictors on patient safety. However, there are several gaps in the literature on patient safety that still need to be addressed. First, much of the empirical research on contextual factors has employed quantitative time-series, before-and-after, or cross-sectional research designs [28]. However, context-related factors such as teamwork and turnover intention are inherently socially constructed phenomenon and greater use of qualitative or mixed methods designs can provide valuable insights that may be missed by over-reliance on quantitative research [29]. Second, past empirical research has focused primarily on certain patient safety predictors – e.g., teamwork – while the impact of other pertinent patient safety predictors – e.g., turnover intention – have largely been underexplored. Third, empirical research in healthcare settings has been limited to an examination of *main effects* of constructs on outcomes with little attention to potentially important *interactive effects* [6, 29] – there is a need to examine mediating and moderating influences of predictors on safety outcomes.

The current mixed-methods study seeks to address the above noted gaps in the patient safety literature by examining how nurses’ perceptions of senior leaders, immediate supervisors, teamwork and turnover intention impact their perceptions of patient safety. More specifically, it is hypothesized that:

- **Hypothesis 1***: Perceptions of senior leadership support for safety, supervisory leadership support for safety, and teamwork will be positively associated with overall patient safety grade. All of these associations are predicted to be significant.*
- **Hypothesis 2:** *Perceptions of turnover intention will be negatively associated with overall patient safety grade. This association is predicted to be significant.*
- **Hypothesis 3:** *The leadership and teamwork predictor variables will moderate the negative impact of turnover intention on perceptions of overall patient safety grade.*

## Methods

### Setting

The current study was conducted at a large community hospital located in Southern Ontario. The hospital has approximately 300 inpatient beds and offers a variety of speciality services including cancer care, cardiac care, paediatrics and mental health services.

### Ethics approval and consent to participate

Ethics approval was obtained from the participating hospital’s Ethics Board and the Human Participants Review Sub-Committee of York University’s Ethics Review Board (Certificate #STU 2016–016). All of the study’s procedures (e.g., recruitment, data collection) were carried out in accordance with the relevant guidelines and regulations. Informed consent was obtained from all of the study participants.

### Overall study design

The current study utilized the concurrent embedded mixed methods design [30] during which a) quantitative survey data and qualitative semi-structured interview data were collected concurrently, in a single phase, over a four-month period (ending in 2016), b) analyses of quantitative survey data and qualitative semi-structured interview data occurred separately, c) mixing of quantitative results and qualitative findings occurred at the discussion stage by the use of methods triangulation to investigate similarities and differences between survey results and interview findings.

### Sampling and data collection procedures

In total, 185 completed surveys were returned and 15 semi-structured interviews were conducted. It was not feasible to acquire accurate staffing numbers from unit managers because part-time and casual staff are assigned to a unit based on need. The results section provides more information on the sample characteristics.

#### Quantitative survey

The survey used in the current study was constructed from previously validated scales [31–35] – detailed information on these scales is provided in the measures section below. Survey data were obtained from frontline nurses (i.e., registered nurses and registered practical nurses) and other non-physician healthcare professionals (e.g., respiratory therapists, physiotherapists, pharmacists) who had worked for at least 6 months on a participating clinical unit: intensive care unit (ICU), general medicine, adult inpatient mental health, or emergency department (ED). Staff with a leadership role (e.g., nurse manager) or those not in direct contact with patients (e.g., unit clerks responsible for administrative duties) were excluded.

Non-probability convenience sampling was used to recruit eligible full-time, part-time, and casual staff. The lead author was responsible for recruitment and survey distribution. A verbal informed consent from each eligible participant was obtained before a survey was handed out. In addition, a returned completed survey constituted a respondent’s consent to participate in the study. As a token of appreciation, a raffle draw for $20 gift card was held on each participating unit.

#### Qualitative semi-structured interviews

A non-probability sampling procedure was utilized to recruit frontline nurses for interviews. The scope and number of semi-structured interviews was limited by logistical and practical reasons (e.g., the hospital’s ethics board granted approval for the study with the understanding that the data collection phase would be completed within a 4-month time period; nurse managers needed assurances that the study would not require too much of a time commitment from frontline clinical staff). Consequently, 15 semi-structured interviews, each lasting approximately 40 min, were conducted with three to five nurses on each of the four participating units.

All semi-structured interviews were conducted on-site at the participating hospital in a private room. Before the start of each interview, the participant was provided with two copies of the consent form – one copy was kept by the participant. The consent form highlighted details of the study (e.g., purpose and procedures), assured confidentiality of the collected data, and provided contact information of the research team. An interview commenced only after the participant has signed the consent forms and received adequate answers to any questions relating to the study. The interviewer took hand-written notes and each session was audio recorded to ensure accuracy and to facilitate subsequent data transcription and analyses. At the end of each interview, a $5 gift card was given to the participant as a small token of appreciation.

### Measures

#### Quantitative measures

A survey was constructed using previously validated scales to assess participants’ perceptions of senior leadership, supervisory leadership, teamwork, turnover intention and overall patient safety (PS) grade – see below for details. Demographic data on profession and gender were also collected.

Senior leadership support for safety and supervisory leadership support for safety were measured using the Canadian Patient Safety Climate Survey (Can-PSCS) [31]. The Can-PSCS is a theory-based instrument that has strong psychometric properties validated by confirmatory factor analysis and is currently being used in health settings as part of Accreditation Canada’s Qmentum Accreditation Program. The senior leadership support for safety scale has four items (e.g., “s*enior management considers patient safety when program changes are discussed*”) and reflects staff perceptions of senior leadership commitment to patient safety. The supervisory leadership scale has two items (e.g., “*my supervisor/manager seriously considers staff suggestions for improving patient safety”)* and reflects staff perceptions of frontline level leadership commitment to patient safety. Senior and supervisory leadership support for safety were both previously shown to have strong internal consistency reliability, α > 0.80 [31].

Staff perceptions of teamwork on their respective unit were measured using the Safety Attitudes Questionnaire teamwork climate scale [32]. This scale has 6 items (e.g., “*the physicians and nurses here work together as a well-coordinated team”*) and was previously shown to have good psychometric properties (e.g., α = 0.78) in acute care settings [32]. The senior leadership, supervisory leadership, and teamwork scales all use a five-point agreement Likert scale (1 = “disagree strongly” to 5 = “agree strongly”).

The turnover intention scale consists of 3 items measuring the behavioural intent of an employee to quit his/her current job (e.g., *“I frequently think of quitting this job”*) [33]. This 3-item turnover intention scale showed good discriminant validity in a confirmatory factor analysis of 45 items on job related attitudes [33]. The Cronbach’s α of the scale was previously shown to be > 0.80 [33, 34]. Each item of the turnover intention scale was measured using a seven-point Likert scale where a higher score indicated a higher likelihood that a person would quit his/her current job.

The overall PS grade was taken from the Agency for Healthcare Research and Quality Surveys on Patient Safety Culture (SOPS) Hospital Survey [35]. The overall patient safety grade has one item that asks respondents to select a letter grade (A = excellent to E = failing) for their clinical unit’s performance on patient safety [35].

The negatively phrased items were reverse coded to ensure that a high score on an item corresponded to a high score on a scale. However, the three negatively phrased turnover intention items were not reverse coded as it made intuitive sense that a high score on this scale corresponded to higher intention to leave. A mean score for each scale was calculated if a respondent answered more than half of the questions associated with that scale. The study scales and their items are provided in Additional file 1.

#### Qualitative measures

An interview guide consisting of open-ended questions with multiple probes was utilized to help keep discussions focused on pre-selected study variables – the interview guide can be found in Additional file 2. These open-ended questions and associated probes solicited nurses’ perceptions of how (a) senior leadership support for safety, (b) supervisory leadership support for safety, and (c) teamwork influence patient safety on their clinical units.

### Analysis

#### Quantitative analysis

All analyses were carried out using SPSS, version 11. Manual double entry of survey data was used to minimize data entry errors [36]. Cronbach’s alpha values were calculated for senior leadership, supervisory leadership, teamwork, and turnover intention to assess the reliability of these scales in the current data set [37, 38].

Simple bivariate analyses (Pearson r) were carried out to assess the strength and significance of relationships among the dependent and non-demographic independent variables. The residual scatter and probability-probability plots for turnover intention were examined to ensure that assumptions of multiple linear regression were met [37, 38].

To test our study hypotheses, hierarchical regression analysis was utilized. Hierarchical regression analysis permits a researcher to examine the unique variance accounted for by a predictor, over and above the variance contributed by independent variables entered earlier in an analysis [39]. Demographic variables are typically good candidates for the first step in a hierarchical regression analysis [40], as they are static variables and should be entered in an analysis before the dynamic variables [39]. Hence, unit affiliation and staff demographic (i.e., gender and profession) dummy variables were placed in block 1 and block 2 of the hierarchical regression analysis, respectively. The four predictors (i.e., senior leadership, supervisory leadership, teamwork, and turnover intention) and their associated interactions were placed in blocks 3 and 4, respectively. All predictors with interactions were centered to avoid problems of multicollinearity [41] and significant interactions were plotted.

#### Qualitative analysis

Two undergraduate research assistants transcribed all the semi-structured interviews, verbatim. Before commencing qualitative data analysis, the primary researcher compared each transcript with the audio-recording of a given interview to confirm the completeness of the data and anonymize the transcripts to protect the privacy and confidentiality of the participants (e.g., each interviewee was given a pseudonym).

The current study relied on typological analysis that was implemented in 6 sequential steps [42, 43]. In *step 1*, initial categories were identified from the study’s hypotheses at the factor levels. In *step 2*, each interview transcript was read completely with only one of the factor level categories (e.g., teamwork) in mind and all places in the data were marked where evidence of this category was present. In *step 3*, data pertaining to each factor were analyzed separately in all transcripts for the discovery of sub-factor level constructs. In *step 4*, each interview transcript was re-read to ensure that the non-coded data did not contain important and/or contradictory information that should either be integrated into existed categories or coded into new categories. In *step 5*, the relationships between factor and subfactor categories and their importance to patient safety were delineated. This process was guided by the information on these relationships from the research literature, study hypotheses, and declarations from interview participants. In *step 6*, direct quotes from nurse interviewees were selected to illustrate the relationships that were discovered in the previous step.

A variety of strategies for trustworthiness were employed including dependability through detailed methodological description, confirmability through an audit trail, and credibility through independent verification of themes and subthemes by members of the research team [44].

## Results

### Quantitative results

#### Response rate and sample characteristics

A total of 185 out of 245 distributed surveys were completed and returned. Four of the eligible clinical staff refused to take a survey and two respondents were excluded as they did not meet the inclusion criteria of the study. The overall survey response rate was 74.1%, range = 67% (ED) to 92% (General Medicine) (see Table 1).

**Table 1:** Survey Response Rate by Clinical Unit

	Distributed	Refused Survey at Handout	Excluded (ineligible)	Returned	Response Rate = (Returned - ineligible) ÷ (Distributed + Refused - ineligible)
Intensive Care Unit	66	2	0	49	49/68 = 72.1%
General Medicine	49	0	0	45	45/49 = 91.8%
Emergency Department	88	1	1	60	59/88 = 67.0%
Mental Health	42	1	1	31	30/42 = 71.4%
Total	245	4	2	185	183/247 = 74.1%

Most study participants were female (89.6%) and nurses (79.8%). The proportion of nurses (79.8%) and other non-physician healthcare professionals (17.5%) respondents was similar to their proportion in participating units’ full-time staff where 82.5% were nurses and 17.5% were other non-physician health professionals – see Table 2. Other demographic data (e.g., proportion of part-time and casual healthcare staff) were not available.

**Table 2:** Demographic Information of the Whole Sample (*N* = 183)

		Frequency		Percent	
Gender	Female	164		89.6	
	Male	16		8.7	
	No response	3		1.6	
	Total	183		100	
Profession^a^	Nurses	146	*264*	79.8	*82.5*
	Other healthcare professionals	32	*56*	17.5	*17.5*
	No response	5	*–*	2.7	*–*
	Total	183	*320*	100	*100*

Note^a^: Professional breakdown of full-time staff reported in italics

#### Bivariate analyses

Table 3 shows results of the bivariate analyses and reveals significant relationships among the predictor and outcome variables. The Cronbach’s α value for the teamwork scale was .78 and α exceeded .80 for the other scales – α values of multi-item scales are shown in the diagonal in Table 3. The lowest mean score was provided for senior leadership support for safety (3.01/5).

**Table 3:** Means, Standard Deviations (SD) and Pearson r Correlations (*N* = 183)

	Mean	SD	1	2	3	4
1. Senior Leadership	3.013	.936	*.87*			
2. Supervisory Leadership	3.613	1.026	.490**	*.82*		
3. Teamwork	3.606	.667	.402**	.593**	*.78*	
4. Turnover Intention	3.206	1.729	−.191*	−.140	−.339**	*.89*
5. Overall PS Grade	3.08	.883	.574**	.469**	.559**	−.351**

Note: *p < .05

**p < .01

#### Hierarchical linear regression analysis

The unit affiliations, staff demographics, predictors, and interactions were entered in block 1, 2, 3, and 4 respectively of the hierarchical regression analysis (see Table 4). Twenty-two percent of variance in overall PS grade was accounted by unit affiliations (*p* < .001). The beta coefficients for intensive care unit (β = .743, *p* < .001) and adult mental health unit (β = −.569, *p* < .01) were significant. The staff demographics did not explain a significant amount of variance in overall PS grade.

**Table 4:** Results of Hierarchical Regression Analysis (DV = Overall PS Grade)

	Model 1, β	Model 2, β	Model 3, β	Model 4, β
*Block 1 – Unit Affiliation*				
ICU	.743***	.735***	.559***	.504***
ED	.172	.182	.132	.072
Mental Health	−.569**	−.559**	−.194	−.185
*Block 2 – Staff Demographics*				
Female		−.272	−.063	−.020
Nurses		−.034	.205	.181
*Block 3 – Predictor Variables*				
Senior Leadership			.417***	.417***
Supervisory Leadership			−.048	−.064
Teamwork			.298**	.264**
Turnover Intention			−.080**	−.081**
*Block 4 – Interactions*				
Senior x Supervisory				−.058
Senior x Teamwork				−.208
Senior x Turnover				−.004
Supervisor x Teamwork				.059
Supervisory x Turnover				.020
Teamwork x Turnover				−.132*
*Total R*^*2*^ *(adjusted)*	.219***	.217	.523***	.539
*Change in R*^*2*^	.233***	.007	.308***	.032

Note: ***p < .001, **p < .01, *p < .05. (*N* = 170). Reference groups: General medicine, Male, and Other non-physician healthcare professionals

**Fig. 1:**
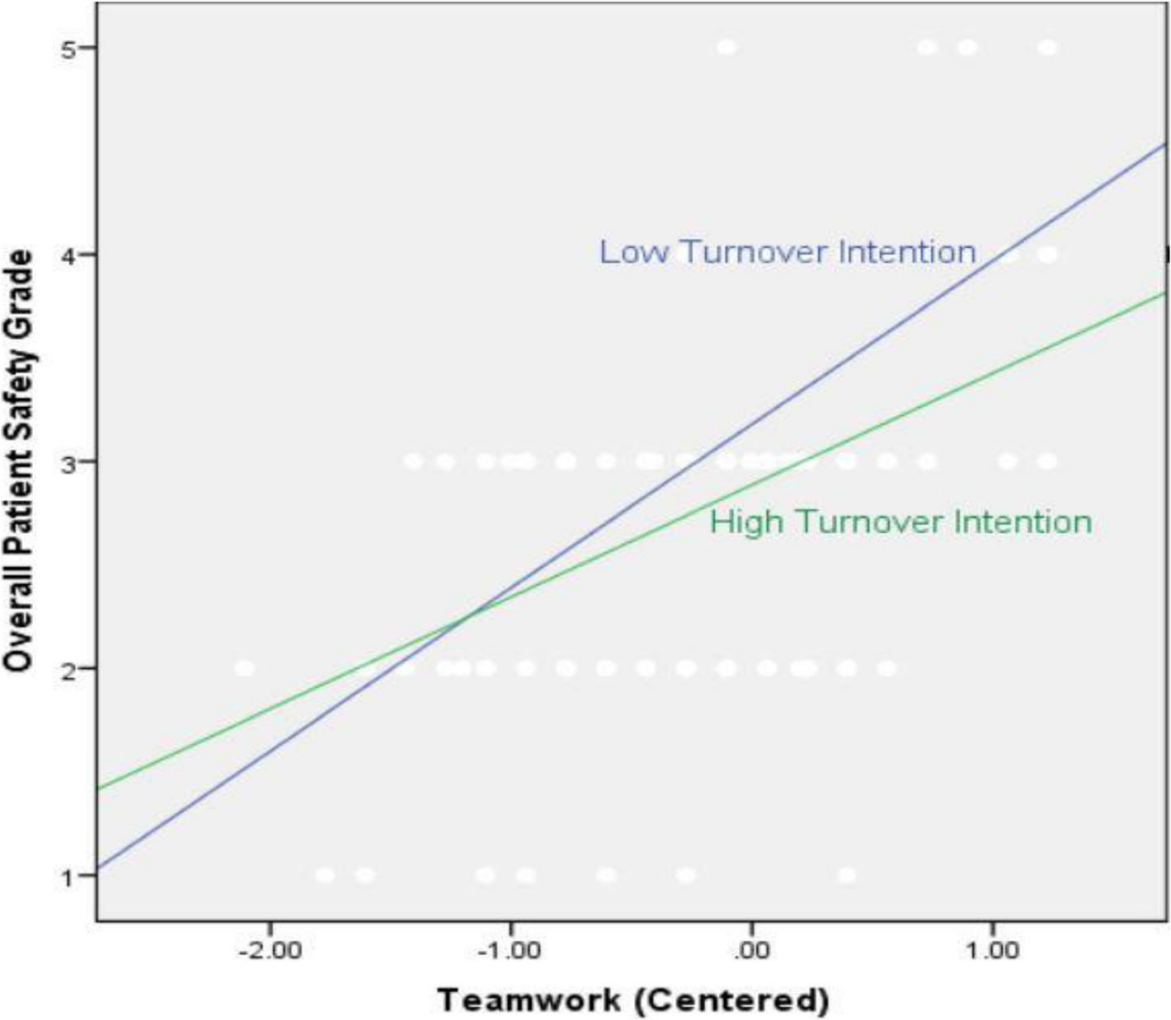
The relationship between teamwork and overall PS grade at different levels of turnover intention

The four predictors explained 31% of variance in overall PS grade (*p* < .001). The beta coefficients for senior leadership support for safety (*p* < .001), teamwork (*p* < .01) and turnover intention (*p* < .01) were significant. Finally, the six interactions did not explain a significant amount of variance in overall PS grade. However, the interaction between teamwork and turnover intention (*p* < .05) was significant. The significant interaction between teamwork and turnover intention is plotted in Fig. 1. This figure highlights the relationship between perceptions of teamwork and overall PS grade when turnover intention is high and when it is low. It shows that when perceptions of turnover intentions are low, the positive impact of teamwork on overall PS grade is significant but perceptions of teamwork become a less important predictor of PS grade if an employee is planning to leave the organization. In total, the regression model accounted for 54% of the variance in overall PS grade.

### Qualitative interview findings

Before the start of data analysis, an initial coding system (Bold Table 5 Themes) was developed from theory and study hypotheses. Subsequently, concepts at the sub-theme level were developed from the data during the coding of interview transcripts (see Table 5).

**Table 5:** Coding Scheme

*Themes*	*Sub-themes*
**Perceptions of senior leadership support for safety**	Relational competency (e.g., visibility)
	Communication with the frontline staff
	Policy making
**Perceptions of supervisory leadership support for safety**	Supervisory leader-staff safety related communication (e.g., supervisory leader’s feedback to reported errors)
	Relational competency (e.g., approachability)
**Perceptions of teamwork** within a unit	Inter-professional teamwork
	Intra-professional teamwork
	Tenure
	Nurse-to-patient ratio / staffing level
**Perceptions of teamwork** across unit boundaries	Inter-professional teamwork
	Intra-professional teamwork

Note: Bolded Categories were developed from theory; non-bolded categories were developed from the data

The qualitative data analyses showed that interviewees perceived a lack of support for safety from senior leaders at all participating clinical units. The nurses were of the view that the hospital’s senior leaders need to be more visible at the frontlines, communicate their vision about the hospital much more clearly and involve frontline clinical staff in policy making discussions.

Interviewees from ED, ICU, and the general medicine unit held positive perceptions of supervisory leadership support for safety – the only exception being the commonly held belief that supervisory leaders rarely provide feedback when minor events or near misses occur. On the other hand, the majority of mental health nurses held negative perceptions of supervisory leadership support for safety. In general, the frontline nurses preferred participative or supportive supervisory leadership style over directive leadership style. A ‘supportive’ supervisory leader was seen as someone who is approachable, values staff expertise, provides timely feedback and is receptive to staff concerns. In contrast, a ‘directive’ supervisory leader was seen as someone who micro-manages day-to-day functioning of the unit and seldom relies on the expertise of the frontline clinical staff while making decisions.

The data analyses revealed that nurses’ perceptions of teamwork are strongly influenced by profession and unit boundaries. Within unit boundaries, the interviewees held positive perceptions of intra-professional teamwork (i.e., nurse-nurse) on all participating clinical units, whereas, staff perceptions of inter-professional teamwork (e.g., nurse-physician, nurse-physiotherapist, nurse-police) were primarily negative on 3 out of 4 clinical units – ICU being the exception where interviewees believed that clinicians belonging to different professions worked well as a team on the unit. Interviewees also suggested that the presence of newer staff or inexperienced staff as well as low nurse-to-patient ratio can compromise patient safety and the quality of teamwork on a clinical unit. Teamwork across unit boundaries was brought up in the context of intra-hospital patient transfers – primarily patient transfers from the ED – and the process was described as “poor” by nurses. Teamwork across clinical unit boundaries was a particular area of concern for interviewees from the adult in-patient mental health unit. They felt that clinical staff from the ED lacked understanding about the complexity of treating mental health patients and were prone to provide incomplete information during patient transfers. Examples of nurse interviewees quotes on leadership and teamwork are presented in Tables 6 and 7 respectively.

**Table 6:** Sample quotes on leadership

*Themes and Sub-themes*	*Sample quotes of nurse interviewees*
Senior leadership’s visibility and communication at frontlines.	I have worked here for many years; I don’t think I have ever met any of the senior management in this hospital or had a chance to discuss anything with them so I feel they could probably come around a lot more to our department, especially when we constantly have bed crises ………. and we are ...... the frontline of the hospital.
Senior leadership’s visibility and communication at frontlines.	There is a lot of, I find horizontal conversations happening but not a lot of top down communication. So, a lot of the feelings from nurses is that senior management have no idea about what is going-on on the floor. A lot of people feel that way because we don’t hear the background, we don’t hear what [the senior leaders] are talking about. We just see the very end result and [senior leaders] just say “hey we are doing this”.……… we don’t hear all the discussions that they are having in senior management meetings.
Senior Leadership and inclusive policy making.	As [senior leaders] are trying to make the unit safe, or the patients safe, they’re not asking for the staff to comment or participate in those meetings where they created [policies] ………. unless you have frontline staff that are most affected by a policy, you’re never going to create a safe policy because you cannot sit in an office and not practice and not deal with the day to day ins and outs of the physical unit and know that that policy is an appropriate policy to implement.
Supervisory leadership and safety related communication.	There is a huddle board that gets done, patient huddle board, for the most part it gets done on daily basis and there the nurse manager discusses what could be safety issues ………. and you feel free to bring up anything you want to in-front of the group, so more suction equipment, sometimes that might be a safety issue. Now, [the unit] has ordered some extra suction equipment so that we can have enough for patients in every room.
Supervisory leadership and relational competency.	I really like my [supervisory leader], she is really approachable, to be honest. I feel comfortable with her ......... the other day we had a patient and I felt like there was a lot of buck passing in his care. He was a homeless man, he couldn’t go back to the shelter, he needed a nursing home. They refused to admit him to the hospital so he was [downstairs] for 5 days which seems a little inappropriate to me and then there were issues like getting his meds from the pharmacy because they only send those for admitted patients and the kitchen wouldn’t send him a warm meal because they only send warm meals for admitted patients. I went to [my supervisory leader] and complained, passed on all my concerns about the patient. She was quite open to my concerns and how to fix what was happening ………... [she] did call the food services and talked to their supervisor and talked to the pharmacy and kind of said, this is the situation and you need to appropriately change polices for this patient.

**Table 7:** Sample quotes on teamwork

*Themes and Sub-themes*	*Sample quotes of nurse interviewees*
Intra-professional teamwork within a unit.	[On this unit] as nurses, we work very well as a team because we understand that it needs to be done as a team because that is the best way that the patient gets taken care of and the best way they feel heard and feel at ease being here.
Inter-professional teamwork within a unit.	I do feel that sometimes there is a lack of …… communication from the physician team and that they don’t communicate what their plan of care is with the nursing staff ……. I find it really helpful when physician say, “ok we have this patient in here, they are this old, they have these comorbidities, I feel that this is a cardiac arrest and we are going to continue the resuscitation, we are going to get pulse check in 2 min”. When the physician speaks about what they are thinking, it just provides a whole lot of clarity for the team and we are not pulling in different directions. Often times the nurses, we are talking to each other, we are planning ahead which is good …… but I feel that the physicians need to take the lead.
Teamwork and tenure.	It is harder for newer staff members coming on to this unit, they don’t know people; they don’t know who they can talk to so, being here for [a few] years, I know who I can talk to and how to get things done that I need to get done and I know if I have concerns whom to talk to but a new staff member I think will probably have a hard time with that because they don’t know anybody so they don’t know who they can talk to which would then be a patient safety concern because they are trying to manage everything on their own and you cannot do that on the floor.
Teamwork and nurse-patient ratio / staffing level.	[Unit name] is where I worked for years previously before coming to this unit. Sometimes up on [my old] unit, where there is a higher ratio of patients and very minimal staff, it was hard to support one another. People got tired and it was just different; the physicality of the job was much heavier… Here [you have more staff]. …… you just have more people if you need to grab people, which is what it should be.
Inter-professional teamwork across unit boundaries.	You got staff downstairs who have no clue what we do up here ……... they don’t get it nor do they want to get it, [for them] everything revolves around [their unit]. You cannot assess somebody in a 20-min period and decide what you want to do with them, it takes some time. They are constantly pushing, pushing, pushing and then when you call them to try to get information, the left hand doesn’t know the right ……. they don’t know the mental health act, they don’t know anything about mental health.
Intra-professional teamwork across unit boundaries.	I was taking in an admission for a patient from [another] department ……. it was a …… nurse who had this patient and she gave me a report, it was pretty brief. She didn’t say very much, she told me that “he is calm and cooperative, nothing is wrong with him”.…… and after I hung up with her, I realized that I didn’t get a very good report so I tried to call back ……... for another report, nobody answered. So, I had to page my manager, she called …….. the [other department] to talk to their manager but in the process of this all happening, the patient arrived [up here] on the unit while I was waiting to get a better report.

## Discussion

The study results only partially supported hypothesis 1. Staff perceptions of senior leadership support for safety were shown to be significantly associated with overall PS grade. These results were corroborated by the qualitative interview findings as nurses believed that senior leaders who prioritize safety through clear communication, inclusive policy making, and high visibility at the frontlines have a positive impact on their perceptions of patient safety.

Survey and interview data on the extent of senior leadership support for safety compared to supervisory level support were consistent – both nurse interviewees and survey respondents, on average, held more positive perceptions of supervisory leadership support for safety compared to senior leadership support for safety (see Table 3 – supervisory leadership’s mean = 3.61 and senior leadership’s mean = 3.01). The survey results showed a non-significant association between supervisory leadership and overall PS grade. However, the interview findings highlight the significance of supportive frontline managers to nurses’ positive perceptions of patient safety. This inconsistency may be simply due to a narrow operationalization of supervisory leadership in the survey where only two *proactive* supervisory safety behaviours were measured – i.e., asking staff for safety improvement suggestions and encouraging them to follow established safety procedures. Nurse interviewees viewed the safety specific role of frontline managers much more broadly – e.g., timely feedback from a nurse manager after an error was seen as a key safety specific supervisory behaviour. In future survey research, a broader operationalization of supervisory leadership support for safety may reveal its significant direct effect on patient safety.

The survey results showed that teamwork was significantly associated with the self-reported perceptions of overall patient safety grade. The qualitative findings corroborated the survey results as nurses perceived positive teamwork on a clinical unit as being critical for patient safety outcomes. However, interview findings suggest that the nurses distinguished teamwork across professional and unit boundaries. In general, the interviewees held positive perceptions of intra-professional teamwork, and negative perceptions of inter-professional teamwork, on a clinical unit. This is consistent with past empirical research that shows the majority of work-related collaboration occurs within professional boundaries whereas tokenistic collaboration is the norm across professional boundaries in healthcare settings [45, 46]. Interestingly, the qualitative findings of the current study suggest that the mental health nurses were especially concerned about the quality of nurse-police teamwork on their clinical unit. The nurses felt that police officers stationed on the unit were not adequately trained on mental health issues and often brushed off nurses’ safety concerns. Indeed, empirical evidence is starting to emerge that suggests law enforcement officers require better training on mental health issues (e.g., psychiatric disorders, effective communication skills) [47] and that emergency crisis teams consisting of closely collaborating police officers and mental health clinicians can improve patient safety outcomes [48].

Our qualitative findings indicated that both higher tenure and higher staffing levels are beneficial for teamwork and patient safety on a clinical unit. This is consistent with previous empirical research showing a higher nurse-to-patient ratio or nurse staffing level is significantly associated with lower medication errors and lower length of stay [49], lower odds of hospital related adverse events (e.g., in-patient mortality, nosocomial bloodstream infection) [50] and better nursing teamwork climate [51]. Similarly, higher nurse tenure or years of experience on a clinical unit has been shown to be significantly associated with lower incidence of patient infections (e.g., pneumonia, pressure ulcers) [52], lower patient’s residual length of stay [53] and better nurse-physician collaboration [54]. The survey results showed that ICU clinical staff held more positive perceptions of overall PS grade and interview findings revealed that the ICU nurse-patient ratio was much higher compared to the other three clinical units. This qualitative finding on nurse-patient ratio provides a potential explanation for the significantly positive association of ICU and overall PS grade.

Our qualitative findings of nurses’ negative perceptions of the teamwork across unit boundaries for intra-hospital patient transfers also add to the literature. Much of the empirical research on patient handoffs or patient transfers has focused on inter-shift handoffs within the same department and inter-hospital patient transfers [55]. The limited empirical research on intra-hospital patient transfer suggests that poor teamwork among clinicians involved in the transfer process can often lead to medical errors and associated patient harm [56, 57]. This is an area that would benefit from further research.

Hypothesis 2 was supported as the survey results showed that lower turnover intention was significantly associated with higher self-reported perceptions of overall patient safety grade. This finding is consistent with past empirical research on patient safety and turnover intention discussed above. Finally, hypothesis 3 was partially supported as only one of the interactions was found to be significant. However, the significant interaction we found between teamwork and turnover intention makes a novel and important contribution to the literature. It suggests that the positive impact of teamwork on patient safety is significantly reduced when employees’ turnover intentions are high. It is possible that high turnover intention reflects serious morale problems that cannot be repaired with teamwork. This is an area that requires further study. A key recommendation of the Institute of Medicine’s watershed report on building a safer healthcare system [58] was to improve inter and intra-professional teamwork and collaboration in healthcare sectors. The findings of the current study suggest that implementation of staff retention strategies – e.g., mindfulness counselling sessions, staff well-being workshops [59] – are also crucial. This line of enquiry is especially relevant for healthcare delivery organizations that are under increasing pressure to deliver more services while ensuring better outcomes at a lower financial cost. Implementation of patient safety interventions is expensive especially when they are introduced at the frontlines in a piecemeal manner. Instead, implementation of interventions that are capable of synergistically addressing various patient safety predictors (e.g., teamwork and turnover intention) would be a more effective strategy.

### Limitations and future research

This is a cross-sectional mixed methods study. Therefore, cause and effect relationships between predictors and outcome cannot be established. Study participants are more likely to provide socially desirable responses to self-reported measures. In order to minimize this particular limitation, the study participants were assured of their confidentiality and that only anonymized data will be reported in any publications [60]. The common method bias is another limitation of the current study as data on predictors and the outcome were collected using the same survey.

Physicians were not included in this study due to practical considerations. The research team was granted access by the hospital’s Ethics Board with an understanding that all of the data would be collected in 4 months. There were only a small number of full-time physicians on two of the participating clinical units during that time. Moreover, it is difficult to recruit physicians as they are often not physically present on a unit throughout a shift. Given these practical considerations we were unable to include physicians as a sub-group in the current study. Their perceptions of contextual variables such as leadership and teamwork are, however, unique and important to the study of patient safety in healthcare settings.

Finally, convenience sampling was used to recruit study participants from a single community hospital. Future research would benefit from more robust sampling procedures. Moreover, collection of predictor and outcome survey data separately would improve the rigor of future research studies. It is also recommended that the findings of the current study are tested in other clinical units (e.g., oncology, radiology), professions (e.g., physicians), and hospital types (e.g., teaching, urban, rural).

### Implications for practice

In the current study, relationship-oriented leadership style was preferred by frontline staff over task-oriented leadership style. In healthcare settings, patient and staff outcomes are positively impacted when leaders engage in relational practices (e.g., providing support for safety and timely feedback) in their interactions with frontline clinical staff [15]. Leadership support for safety is a modifiable contextual factor [61] that can be built and strengthened as part of patient care improvement interventions [62].

Healthcare employees are more likely to hold negative perceptions of patient safety if the quality of teamwork on their clinical unit is poor. This is because poor teamwork leads to subpar patient care and negatively impacts staff well-being. Therefore, healthcare organizations are encouraged to provide on-site continuous educational opportunities designed to foster competencies necessary for effective inter and intra-professional teamwork – e.g., respectful negotiation, conflict resolution, and communication [63].

The current study’s results suggest that the positive impact of teamwork on patient safety is enhanced when staff turnover intentions are low. It is recommended that healthcare organizations proactively implement staff retention strategies. Finally, nursing staff on the mental health unit held poorer perceptions of patient safety and its predictors compared to staff on the other clinical units. Indeed, mental health have been noted for resource scarcity, quality issues, and inadequate staffing levels [64]. There is a need to devote more resources for improving patient safety on mental health units.

## Conclusion

The “*To Err is Human*” [58] report highlighted acute quality and safety deficiencies in the healthcare delivery systems and in doing so energized the scientific community and healthcare professionals to design, evaluate, and implement safety improvement strategies at the frontlines. Indeed, implementation of standardized clinical interventions (e.g., hand hygiene guidelines and surgical checklists) have reduced occurrence of medical errors and associated patient harm. There is increasing empirical support that contextual factors (e.g., teamwork, safety climate) are key determinants of the success or failure of patient safety improvement initiatives; however, certain literature gaps still remain including an over-reliance on quantitative research [28] and limited empirical evidence of potentially important *interactive effects of* patient safety predictors [6].

The survey results of the current mixed methods study suggested that senior leadership, teamwork, and turnover intention demonstrably impact frontline clinical staff perceptions of patient safety. The qualitative findings corroborated the survey results while also providing important insights into why certain statistical relationships may have found to be non-significant (i.e., nurse interviewees perceived the safety specific responsibilities of frontline supervisors much more broadly compared to the narrower conceptualization of the construct in the survey).

A particularly noteworthy finding of the current study was that it highlighted the underexplored but important interactive effect of teamwork and turnover intention on patient safety. More specifically, the positive impact of teamwork on frontline staff perceptions of patient safety would be enhanced if steps are taken to also lower staff turnover intentions. The findings of the current study, together with future research will broaden our understanding of how context influences patient safety and ideally help improve delivery of patient care at the frontlines.

## Supplementary Information

**Additional file 1.** Scales & Associated Items.

**Additional file 2.** Interview Guide.

## Data Availability

The dataset used during the current study is available from the corresponding author, SZ, on reasonable request.

## Abbreviations

PS: patient safety
ICU: intensive care unit
ED: emergency department
Can-PSCS: Canadian Patient Safety Climate Survey
SOPS: Surveys on Patient Safety Culture
