# Supplementary material for: Acute care nurses’ perceptions of leadership, teamwork, turnover intention and patient safety – a mixed methods study
Source: BMC Nurs. 2021 Jul 30;20:134. doi: 10.1186/s12912-021-00652-w (PMC8323271; doi:10.1186/s12912-021-00652-w)
Supplement: Supplementary file 1 — Additional file 1. Scales & Associated Items. [file 12912_2021_652_MOESM1_ESM.docx]

Acute care nurses’ perceptions of leadership, teamwork, turnover intention and patient safety – a mixed methods study

Dr. Shahram Zaheer

PhD

School of Health Policy and Management

York University, Toronto, Canada

Daphne Cockwell School of Nursing

Ryerson University, Toronto, Canada

Lawrence S. Bloomberg Faculty of Nursing

University of Toronto, Toronto, Canada

Email: szaheer@yorku.ca

Dr. Liane Ginsburg

PhD, Professor

School of Health Policy and Management

York University, Toronto, Canada

Email: lgins@yorku.ca

Dr. Hannah J Wong

PhD, Associate Professor

School of Health Policy and Management

York University, Toronto, Canada

Email: hjwong@yorku.ca

Dr. Kelly Thomson

PhD, Associate Professor

School of Administrative Studies

York University, Toronto, Canada

Email: thomsonk@yorku.ca

Lorna Bain

OT Reg. (Ont.)

Interprofessional Collaboration and Education

Southlake Regional Health Centre

Newmarket, Canada

Lecturer, University of Toronto, Canada

Email: LBain@southlakeregional.org

Dr. Zaev Wulffhart

MBBCh., FRCP, FACC

Physician Leader, Regional Cardiac Care Program

Director of Medical Education

Southlake Regional Health Centre

Newmarket, Canada

Assistant Professor, University of Toronto

Email: ZWulffhart@southlakeregional.org

# **Additional File 1: Scales & Associated Items**

**Senior Leadership Support for Safety**

We are looking for your perceptions and opinions of these safety issues. While thinking about your unit, indicate the extent to which you agree or disagree with each of the following statement.

|  | **Strongly Disagree** | **Disagree** | **Neutral** | **Agree** | **Strongly Agree** | **N/A** |
| --- | --- | --- | --- | --- | --- | --- |
| 1. Senior management has a clear picture of the risk associated with patient care | ❒ | ❒ | ❒ | ❒ | ❒ | ❒ |
| 1. Patient safety decisions are made at the proper level by the most qualified people | ❒ | ❒ | ❒ | ❒ | ❒ | ❒ |
| 1. Senior management provides a climate that promotes patient safety | ❒ | ❒ | ❒ | ❒ | ❒ | ❒ |
| 1. Senior management considers patient safety when program changes are discussed | ❒ | ❒ | ❒ | ❒ | ❒ | ❒ |

**Supervisory Leadership Support for Safety**

We are looking for your perceptions and opinions of these safety issues. While thinking about your unit, indicate the extent to which you agree or disagree with each of the following statement.

|  | **Strongly Disagree** | **Disagree** | **Neutral** | **Agree** | **Strongly Agree** | **N/A** |
| --- | --- | --- | --- | --- | --- | --- |
| 1. My supervisor/manager says a good word when he/she sees a job done according to established patient safety procedures | ❒ | ❒ | ❒ | ❒ | ❒ | ❒ |
| 1. My supervisor/manager seriously considers staff suggestions for improving patient safety | ❒ | ❒ | ❒ | ❒ | ❒ | ❒ |

**Teamwork**

Please think about the unit where you work most when responding to the following statements.

|  | **Strongly Disagree** | **Disagree** | **Neutral** | **Agree** | **Strongly Agree** | **N/A** |
| --- | --- | --- | --- | --- | --- | --- |
| 1. It is easy for personnel in this unit to ask questions when there is something that they do not understand. | ❒ | ❒ | ❒ | ❒ | ❒ | ❒ |
| 1. I have the support I need from other personnel to care for patients. | ❒ | ❒ | ❒ | ❒ | ❒ | ❒ |
| 1. Team input is well received in this unit. | ❒ | ❒ | ❒ | ❒ | ❒ | ❒ |
| 1. In this unit, it is difficult to Speak Up if I perceive a problem with patient care. | ❒ | ❒ | ❒ | ❒ | ❒ | ❒ |
| 1. Disagreements in this unit are resolved appropriately (i.e., not *who* is right, but *what* is best for the patient). | ❒ | ❒ | ❒ | ❒ | ❒ | ❒ |
| 1. The physicians and nurses and other team members here work together as a well-coordinated team. | ❒ | ❒ | ❒ | ❒ | ❒ | ❒ |

**Turnover Intention**

Please think about the unit where you work most when responding to the following statements.

|  | **Strongly Disagree** | **Disagree** | **Somewhat Disagree** | **Neutral** | **Somewhat**  **Agree** | **Agree** | **Strongly Agree** |
| --- | --- | --- | --- | --- | --- | --- | --- |
| 1. There is a good chance that I will leave this job in the next year or so | ❒ | ❒ | ❒ | ❒ | ❒ | ❒ | ❒ |
| 1. I frequently think of quitting this job | ❒ | ❒ | ❒ | ❒ | ❒ | ❒ | ❒ |
| 1. I will probably look for a new job in the next year | ❒ | ❒ | ❒ | ❒ | ❒ | ❒ | ❒ |

**Overall Patient Safety Grade**

Please give your work area/unit in this hospital an overall grade on patient safety.

| 🞎 | 🞎 | 🞎 | 🞎 | 🞎 |
| --- | --- | --- | --- | --- |
| **A**  Excellent | **B**  Very Good | **C**  Acceptable | **D**  Poor | **E**  Failing |
