# Supplementary material for: Acute care nurses’ perceptions of leadership, teamwork, turnover intention and patient safety – a mixed methods study
Source: BMC Nurs. 2021 Jul 30;20:134. doi: 10.1186/s12912-021-00652-w (PMC8323271; doi:10.1186/s12912-021-00652-w)
Supplement: Supplementary file 2 — Additional file 2. Interview Guide. [file 12912_2021_652_MOESM2_ESM.docx]

Acute care nurses’ perceptions of leadership, teamwork, turnover intention and patient safety – a mixed methods study

Dr. Shahram Zaheer

PhD

School of Health Policy and Management

York University, Toronto, Canada

Daphne Cockwell School of Nursing

Ryerson University, Toronto, Canada

Lawrence S. Bloomberg Faculty of Nursing

University of Toronto, Toronto, Canada

Email: szaheer@yorku.ca

Dr. Liane Ginsburg

PhD, Professor

School of Health Policy and Management

York University, Toronto, Canada

Email: lgins@yorku.ca

Dr. Hannah J Wong

PhD, Associate Professor

School of Health Policy and Management

York University, Toronto, Canada

Email: hjwong@yorku.ca

Dr. Kelly Thomson

PhD, Associate Professor

School of Administrative Studies

York University, Toronto, Canada

Email: thomsonk@yorku.ca

Lorna Bain

OT Reg. (Ont.)

Interprofessional Collaboration and Education

Southlake Regional Health Centre

Newmarket, Canada

Lecturer, University of Toronto, Canada

Email: LBain@southlakeregional.org

Dr. Zaev Wulffhart

MBBCh., FRCP, FACC

Physician Leader, Regional Cardiac Care Program

Director of Medical Education

Southlake Regional Health Centre

Newmarket, Canada

Assistant Professor, University of Toronto

Email: ZWulffhart@southlakeregional.org

# **Additional file 2: Interview Guide**

**Face-sheet**

**Date of Interview:**

**Time of Interview:**

**Location of Interview:**

**Written Consent Obtained:**

**Extra notes:**

**Interview Guide**

1) Can you tell me about what you do here? For how long have you worked at this unit?

2) Can you think about the last patient safety incident you were involved in? Can you briefly describe what happened?

- Potential probes:
- Is this a typical type of safety issue at your unit?

3) So, what was the outcome of this incident and in your view what factors contributed to this outcome?

- Potential probes:
- a) What about:
- Role of senior leaders?
- Role of supervisors
- Quality of teamwork & communication?

4) Is there anything else we have not discussed yet that you believe is important for delivering safe care at your work place?

**Post Interview Comment Sheet**

**Description of the setting and the participant:**

**Emotional tone of the interview:**

**Any particular difficulties encountered during the interview:**

**My feelings during and about the experience:**

**Personal insights and reflections:**

**Extra notes:**
